# Genetic studies in the Pakistani population reveal novel associations with ventricular septal defects (VSDs)

**DOI:** 10.1186/s12887-023-03851-3

**Published:** 2023-02-09

**Authors:** Sumbal Sarwar, Shabana, Khadija Sajjad, Shahida Hasnain

**Affiliations:** Institute of Microbiology and Molecular Genetics, University of the Punjab, 54590 Lahore, Pakistan

**Keywords:** Ventricular septal defects VSD, Genetic risk score, Pakistani Population

## Abstract

**Background:**

With prevalence up to 4%, Ventricular Septal Defect (VSD) is one of the leading causes of neonatal deaths. VSD is a common complex genetic disorder that has been associated with many genetic determinants. Variants from genes for the transcription factors including T-Box *TBX5* and *NFATc1* (nuclear factor of activated T cells, cytoplasmic 1), Vascular endothelial growth factor (*VEGF*), ISLET1 (encoded by the *ISL1* gene) and enzyme *MTHFR*, a methylene tetrahydrofolate reductase were selected. Genetic risk score (GRS) is a widely accepted approach used to convert the genetic data into prediction and assessment tool for disease susceptibility.

**Methods:**

A total of 200 participants were recruited for the current study, 100 VSD patients and 100 controls. Genotyping of the *ISL1*: rs1017, *NFATc1*: rs7240256, *VEGF*: rs36208048, *TBX5*: rs11067075, and *MTHFR*: rs1801133 variants was performed using tetra primer ARMS PCR and PCR-RFLP. For the statistical analysis, the software SPSS version 23 was used. Genotypic frequencies of cases and controls were calculated using chi-square (χ²) whereas allelic frequencies were calculated by using the SNPStats tool. The association of GRS quartiles with VSD was examined using binary logistic regression. Adjusted *p*-value 0.01 was used as significance threshold for all analyses.

**Results:**

The *ISL1* (OD: 0.242, CI: 0.158–0.37, *p-*value: 2.15 × 10^− 4^ :), *NFATc1* (OD: 2.53, CI: 1.64–3.89, *p-*value: 2.11 × 10^− 5^), *TBX5* (OD: 2.24, CI: 1.47–3.41, *p-*value:1.6 × 10^− 4^) and *MTHFR* (OD: 10.46, CI: 5.68–19.26, *p-*value: 2.09 × 10^− 9^:) variants were found to be in association with VSD. In contrast, the *VEGF* (OD: 0.952, CI: 0.56–1.62, *p-*value: 0.8921) variant did not show significance association with the VSD. For cases, the mean GRS score was 3.78 ± 1.285 while in controls it was 2.95 ± 1.290 (*p-*value: 0.479, CI: 0.474–1.190). Comparison of GRS between cases and control showed that mean GRS of cases was 1.90 ± 0.480 while in controls it was 1.68 ± 0.490 (*p-*value: 0.001, CI: 0.086–0.354). Higher quartiles were more prevalent in cases whereas lower quartiles were more prevalent in controls.

**Conclusion:**

GRS of these five loci was strongly associated with VSD. Moreover, genetic risk score can provide better information for the association between variants and disease as compared to a single SNP. We also illustrated that the cumulative power of GRS is greater over the single SNP effect. This is a pilot scale study with a relatively small sample size whose findings should be replicated in a larger sample size for the unique local Pakistani population.

**Supplementary Information:**

The online version contains supplementary material available at 10.1186/s12887-023-03851-3.

## Background

Millions of genetic markers associated with human disease have been identified by Genome Wide Association Studies (GWAS) over the past decade. However, use of this data in clinical assessment and prediction of disease is still under development. Genetic risk score (GRS) is a commonly used approach to convert the genetic data into prediction and assessment of disease susceptibility. GRS calculates the aggregate of all risk alleles contributing to a genetic disease [1]. Although degree of risk or protection varies depending on the contribution of the number of genetic loci as other environmental factors also have strong influence in disease susceptibility, the approach is still helpful [1].

As GRS compiles the results of multiple variants in a quantitative manner, it is preferable over the predictive test of a Single Nucleotide Polymorphism (SNP). GRS can be built by using less than 10 and more than 100 loci [2]. GRS has become a popular tool for cumulative results of SNPs. GRS has already been used in complex diseases as such cardiovascular disease [3, 4]. Ventricular Septal Defect (VSD) is one of the leading causes of neonatal deaths with prevalence up to 4%. During the early fetal development, the ventricular septum of the heart fails to develop properly and leaves a gap resulting in VSD [5]. This is a cardiac malformation which can manifest as a single anomaly as well as with a combination of other disorders [6]. VSD is a common complex genetic disorder that has been associated with many genetic determinants along with environmental factors [7]. Asia has the highest prevalence of CHD (9.3/1000 live births) and VSDs (2.62/1000 live births). In Pakistan, 30% of patients diagnosed with CHD have VSD [6]. Several studies on heart disorders have been conducted using GRS method in Pakistani population [2, 8].

**Fig. 1:**
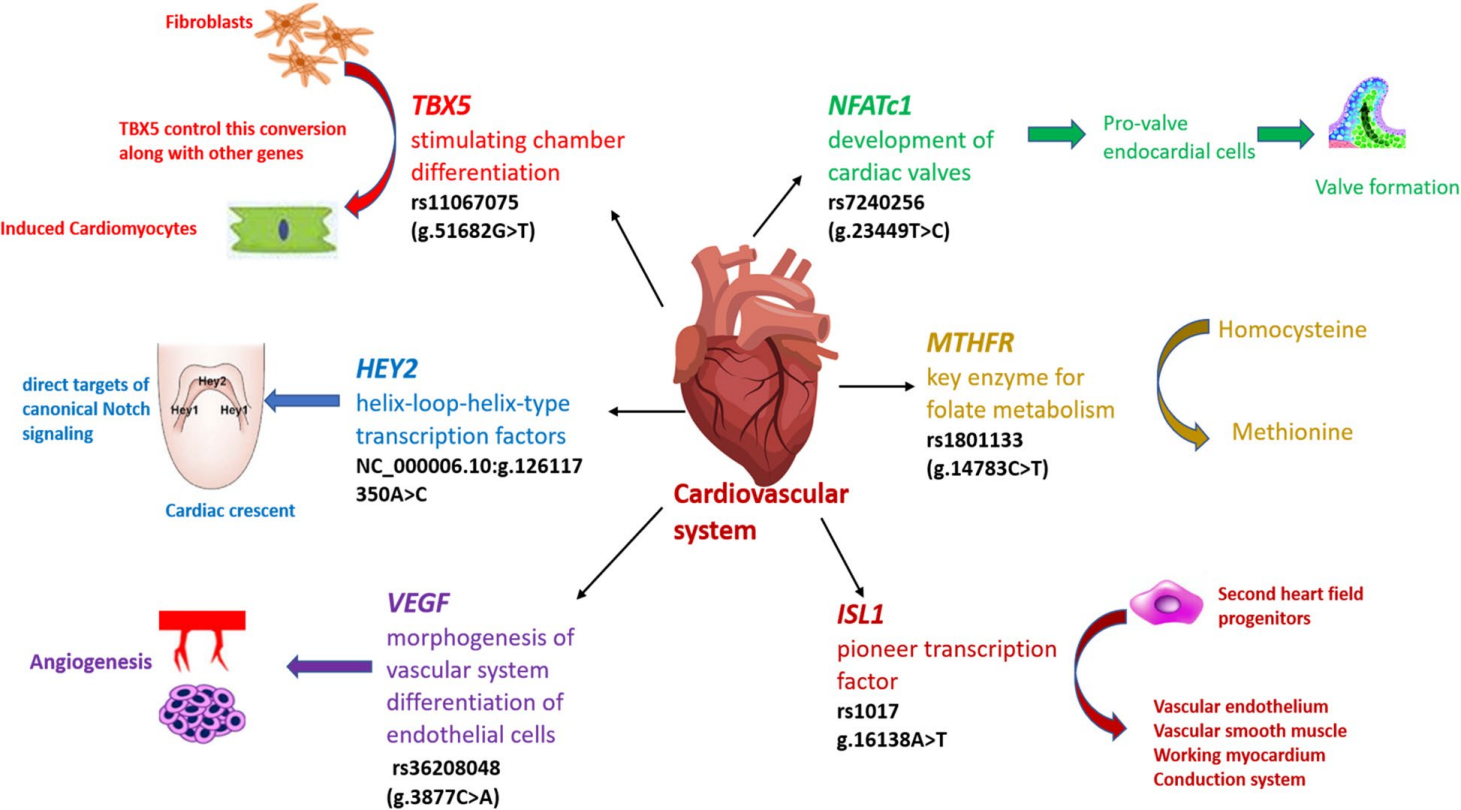
Comparison of cumulative genetic risk score among cases and controls

*ISL1* is a LIM homeodomain transcription factor expressed transiently in the second heart field and serves as a marker for progenitor cells [9]. *ISL1* is present on chromosome 5q11.1. Studies have reported genetic variation in Nuclear Factor of Activated T Cells 1 *NFATc1* as the cause of atrio-ventricular septal defects (AVSD). The human *NFATc1* gene is located on chromosome 18q23. [10]. Vascular endothelial growth factor (*VEGF*) is an angiogenic regulator and signaling protein involved in the formation of blood vessels. *VEGF* gene is located on chromosome 6p21.1. Also plays a key role in morphogenesis of the vascular system and differentiation of endothelial cells [11]. Many transcription factors (e.g. NKX2.5, GATA4, *TBX5*) and signaling pathway molecules are involved in normal heart development. T-Box transcription factor *TBX5* is primarily known for cardiac and forelimb development. *TBX5* gene is located on chromosome 12q24.21. It also acts as transcriptional activator for the genes specifically associated with cardiomyocyte maturation [12]. *MTHFR*, a methylene tetrahydrofolate reductase, is an enzyme which makes the initial circulatory form of folate which in turn acts as a donor of methyl group for re-methylation of homocysteine to methionine. The human *MTHFR* gene is located on chromosome 1p36.22. [13] (Fig. 1).

To date a large number of genetic association studies have been conducted on European populations. Most scientists compare the results of those studies with the rest of the world. But there is a dire need to broaden the horizon of genetics studies in other ethnicities because different ethnic groups have a varied genetic makeup. The genetic architecture of the Pakistani population is unique. Like other Asian countries, this population is also under-represented in the 1000 genome project with only 92 participants [14]. The current study is a pilot scale study to explore GRS in relation to VSD. In this study, five variants in the *ISL1*: rs1017 (NG_023040.1:g.16138A > T, 3 prime UTR Variant), *NFATc1*: rs7240256 (NG_029226.1:g.23449 T > C, intron Variant), *VEGF*: rs36208048 (NG_008732.1:g.3877C > A, 5’ upstream Variant), *TBX5*: rs11067075 (NG_007373.1:g.51682G > T, regulatory region variant), and *MTHFR*: rs1801133 (NG_013351.1:g.14783C > T, coding region variant) genes are investigated in order to construct GRS in Pakistani VSD patients. The aim of the present study was to recruit Pakistani VSD patients to determine the association of GRS of selected genetic variants with VSDs. The null hypothesis of the study is that the predictive value of GRS was not superior over the single SNP effect size.

## Methods

### Ethical approval, written consent and recruitment of study subjects

A total of 200 participants were recruited for the current study, 100 VSD patients and 100 controls. VSD patients were selected from the various cardiac units of different hospitals. Healthy controls with no cardiac defects were included in this study. Written and verbal consent was taken from parents or guardians of participants. The study was approved from the local ethics committee of University of the Punjab, Institute of Microbiology and Molecular (Ref. no #MMG 1958–13/10/2020). The new children referred to a tertiary referral centers below 15 years of age were included in the study. The diagnosis was made based on echocardiography. Size, number and exact location of the defect as well as magnitude of shunt were identified by two dimensional and Doppler echocardiography. The samples having VSD with other cardiac or extra-cardiac symptoms (syndromic VSD) and any seropositive sample (for HBV/HCV/HIV) in both cases and controls were excluded from the study as described previously [6, 15, 16].

### Genotyping

The pediatric blood samples were taken in EDTA vials and preserved at -20 °C. Genomic DNA from the human leukocytes/epithelial cells was isolated using the salting out method (see supplementary data for salting out method). The quality of extracted DNA was evaluated using an Epoch Biotek micro-plate reader (Biotek Instruments, USA) and DNA concentration was adjusted to 10 ng/uL. The SNPs of *ISL1*: rs1017 (NG_023040.1:g.16138A > T), *NFATc1*: rs7240256 (NG_029226.1:g.23449 T > C), *VEGF*: rs36208048 (NG_008732.1:g.3877C > A), *TBX5*: rs11067075 (NG_007373.1:g.51682G > T), and *MTHFR*: rs1801133 (NG_013351.1:g.14783C > T) genes were genotyped by using tetra primer the ARMS PCR and PCR–RFLP techniques. For primers sequence and restriction enzyme, see supplementary data (supplementary table 1).

### Construction of genetic risk score

For construction of the gene score, the risk alleles of all SNPs were considered to be acting in an additive manner. Each risk allele was supposed to have an equivalent contribution to the outcome, so each risk allele was coded as 1. The protective homozygous genotype with no risk allele was coded 0, the heterozygous individual with one risk and one normal allele was coded as 1 and the risk homozygous individual with both risk alleles was coded as 2. In this way the GRS of an individual can range from 0 (no risk allele) to 10 (with all risk alleles for 5 loci) [2, 8].

### Statistical analysis

For the statistical analysis, the software SPSS version 23 was used. Genotypic frequencies of cases and controls were calculated via chi-square (χ^2^) whereas allelic frequencies were calculated by using the SNPStats web tool. The distribution of GRS in cases and controls was compared by histograms. To compare means of GRS in cases and controls, an independent sample t-test was performed. To examine how well GRS discriminates between VSD cases and controls, the receiver operating curve (ROC) was analyzed. The ROC curve is a graph of sensitivity against specificity and was plotted by selecting GRS and VSD status as state variables. The effect of a range of risk alleles was calculated by GRS quartiles selecting the lowest through highest GRS levels. The association of the GRSs quartiles with VSD was examined using binary logistic regression by selecting GRS as covariates and VSD status as dependent variable. Due to the inclusion of 5 SNPs, a Bonferoni adjusted *p*-value of 0.01 was used as significance threshold for all analyses.

## Results

For the rs1017 (g.16138A > T) variant in *ISL1*, the genotype frequencies were of 52% (AA), 11% (TA) and 37% (TT) in cases in comparison to 21%, 8% and 71% respectively in controls (Table 1). The frequency of the T and A alleles is 0.42 and 0.58 respectively in cases, while 0.75 and 0.25 respectively, in controls (OR: 0.242, CI: 0.158–0.37, *p-value* < 0.0001). Genotyping of rs36208048 (g.3877C > A), variant in the *VEGF* gene showed that the percentage of the AA, CA and CC genotypes were 0%, 32% and 68% respectively in cases versus 0%, 33% and 67% in controls (Table 1). The frequency of the C and A alleles were 0.84 and 0.16 respectively in cases and 0.83 and 0.17 in controls (*p-value* 0.8577). In case of rs7240256 (g.23449 T > C) *NFATc1* variant the genotype frequencies were 4% (CC), 78% (TC) and 18% (TT) in cases compared to 1.0%, 44% and 55% in controls (Table 1). The minor allele frequency (MAF) was 0.43 in cases while 0.23 in controls (OR: 2.53, CI: 1.64–3.89, *p*-value < 0.0001).

**Table 1:** Comparison of genotypic and allelic frequencies between cases and controls

SNPs	Gene	Cases	Controls	*p* value
rs1017: NG_023040.1:g.16138A > T	*ISL1*	AA = 0.52 TA = 0.11 TT = 0.37	AA = 0.21 TA = 0.08 TT = 0.71	2.15 × 10–4
		**T = 0.42** **A = 0.58**	**T = 0.75** **A = 0.25**	** < 0.0001**
rs36208048: NG_008732.1:g.3877C > A	*VEGF*	CC = 0.68 CA = 0.32 AA = 0.00	CC = 0.67 CA = 0.33 AA = 0.00	0.8921
		**C = 0.84** **A = 0.16**	**C = 0.84** **A = 0.16**	**0.8577**
rs7240256: NG_029226.1:g.23449 T > C	*NFATc1*	CC = 0.04 CT = 0.78 TT = 0.18	CC = 0.01 CT = 0.44 TT = 0.55	2.11 × 10^–5^
		**T = 0.57** **C = 0.43**	**T = 0.77** **C = 0.33**	** < 0.0001**
rs11067075 NG_007373.1:g.51682G > T	*TBX5*	GG = 0.21 GT = 0.70 TT = 0.09	GG = 0.56 GT = 0.36 TT = 0.08	1.6 × 10^–4^
		**G = 0.56** **T = 0.44**	**G = 0.74** **T = 0.26**	**2.0 × 10**^**–4**^
rs1801133: NG_013351.1:g.14783C > T	*MTHFR*	CC = 0.25 CT = 0.62 TT = 0.13	CC = 0.88 CT = 0.10 TT = 0.02	2.09 × 10^–9^
		**C = 0.56** **T = 0.44**	**C = 0.93** **T = 0.07**	** < 0.0001**

For the polymorphism, rs11067075 (g.51682G > T) in *TBX5*, the frequencies of the GG, GT and TT genotypes were 21%, 70% and 9%, respectively in cases, versus 56%, 36% and 8% in controls (Table 1). The frequency of the T and G alleles was 0.44 and 0.56 respectively in cases, while 0.26 and 0.74 in controls (OR: 2.24, CI: 1.47–3.41 (*p-value* 0.0002). The genotyping of rs1801133 (g.14783C > T) variant in *MTHFR* showed that the frequencies of the CC, CT and TT genotypes were 25%, 62% and 13%, respectively in cases, while 88%, 10% and 2% in controls (Table 1). The allelic frequency of T and C are 0.56 and 0.44 respectively, in cases, while 0.93 and 0.07 in controls (OR: 10.46, CI: 5.68–19.26, *p-value* (< 0.0001). In case of rs7240256, rs11067075 and rs1801133 for the control group the genotype frequencies were in Hardy–Weinberg equilibrium (*p*-value: 0.021, 0.60 and 0.65 respectively). On the other hand, the genotype frequencies deviated from Hardy–Weinberg equilibrium for the cohort in case of rs1017 and rs36208048 (*p*-value: < 0.0001 and 0.066 respectively). For the dominant model and recessive model the OR, CI and *p-value* mentioned in Table 2. The *ISL1* (OD: 0.242, CI: 0.158–0.37, *p-*value: 0.158–0.37:), *NFATc1* (OD: 2.53, CI: 1.64–3.89, *p-*value: 2.11 × 10^–5^), *TBX5* (OD: 2.24, CI: 1.47–3.41, *p-*value:1.6 × 10^–4^) and *MTHFR* (OD: 10.46, CI: 5.68–19.26, *p-*value: 2.09 × 10^–9^:) variants were found to be in association with VSD in the Pakistani pediatric cohort whilst the *VEGF* (OD: 0.952, CI: 0.56–1.62, *p-*value: 0.8921) variants did not appear to be in association with the VSD (Table 3).

**Table 2:** Dominant and recessive model analysis of the allelic frequencies of SNPs in this study

SNP (Gene)	Model	Genotype	Cases	Controls	OR (CI)	*P*-value
**rs1017 (ISL1)**	Dominant	T/T	37 (36.6%)	71 (71%)	0.21 (0.11–0.39)	< 0.0001
		A/T-A/A	64 (63.4%)	29 (29%)	1.00	
	Recessive	T/T-A/T	48 (47.5%)	79 (79%)	0.24 (0.13–0.43)	< 0.0001
		A/A	53 (52.5%)	21 (21%)	1.00	
	Alleles	T	85 (0.42)	150 (0.75)	0.242 (0.158–0.37)	< 0.0001
		A	117 (0.58)	50 (0.25)		
**rs36208048 (VEGF)**	Dominant	C/C	69 (68.3%)	67 (67%)	1.00	0.84
		C/A	32 (31.7%)	33 (33%)	1.06 (0.59–1.92)	
	Alleles	C	170 (0.84)	167 (0.84)	0.952(0.56–1.62)	0.8577
		A	32 (0.16)	33 (0.16)		
**rs7240256 (NFATc1)**	Dominant	T/T	18 (17.8%)	55 (55%)	1.00	< 0.0001
		T/C–C/C	83 (82.2%)	45 (45%)	0.18 (0.09–0.34)	
	Recessive	T/T-T/C	97 (96%)	99 (99%)	1.00	0.16
		C/C	4 (4%)	1 (1%)	0.24 (0.03–2.23)	
	Alleles	T	115(0.57)	154(0.77)	2.53(1.64–3.89)	< 0.0001
		C	87 (0.43)	46(0.23)		
**rs11067075 (TBX5)**	Dominant	G/G	21 (20.8%)	56 (56%)	1.00	< 0.0001
		G/T-T/T	80 (79.2%)	44 (44%)	0.21 (0.11–0.38)	
	Recessive	G/G-G/T	92 (91.1%)	92 (92%)	1.00	0.82
		T/T	9 (8.9%)	8 (8%)	0.89 (0.33–2.40)	
	Alleles	G	113(0.56)	148(0.74)	2.24(1.47–3.41)	0.0002
		T	89(0.44)	52(0.26)		
**rs1801133 (MTHFR)**	Dominant	C/C	25 (24.8%)	88 (88%)	1.00	< 0.0001
		C/T-T/T	76 (75.2%)	12 (12%)	0.04 (0.02–0.10)	
	Recessive	C/C–C/T	88 (87.1%)	98 (98%)	1.00	0.002
		T/T	13 (12.9%)	2 (2%)	0.14 (0.03–0.63)	
	Alleles	C	113 (0.56)	186(0.93)	10.46(5.68–19.26)	< 0.0001
		T	89 (0.44)	14(0.07)

**Table 3:** Odds ratio, Confidence Interval CI and p-value for selected SNPs

SNPs	Gene	Odds Ratio	95% CI	*p* value
rs1017: NG_023040.1:g.16138A > T	*ISL1*	0.242	0.158–0.37	2.15 × 10–4
rs36208048: NG_008732.1:g.3877C > A	*VEGF*	0.952	0.56–1.62	0.8921
rs7240256: NG_029226.1:g.23449 T > C	*NFATc1*	2.53	1.64–3.89	2.11 × 10^–5^
rs11067075 NG_007373.1:g.51682G > T	*TBX5*	2.24	1.47–3.41	1.6 × 10^–4^
rs1801133: NG_013351.1:g.14783C > T	*MTHFR*	10.46	5.68–19.26	2.09 × 10^–9^

**Fig. 2:**
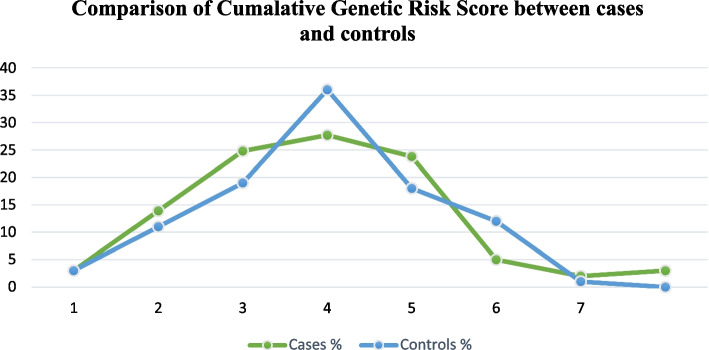
Receiver operator curve, area under ROC=0.668 (C.I=0.593-0.742,  *P*<0.001)

We observed normal distribution of GRS in the whole sample set including cases and controls. A GRS curve (green line) exhibited a shift to the right with higher GRS value for the cases, while for the controls the GRS curve (blue line) exhibited a left shift with lower GRS value. The more prevalent GRS in cases were 3, 4 and 5 with 25%, 28% and 24% respectively while in controls the most prevalent GRS were 3, 4 and 5 with 19%, 36% and 18% respectively. Higher GRS were more prevalent in cases whereas lower GRS were more prevalent in controls (Fig. 2).

**Fig. 3:**
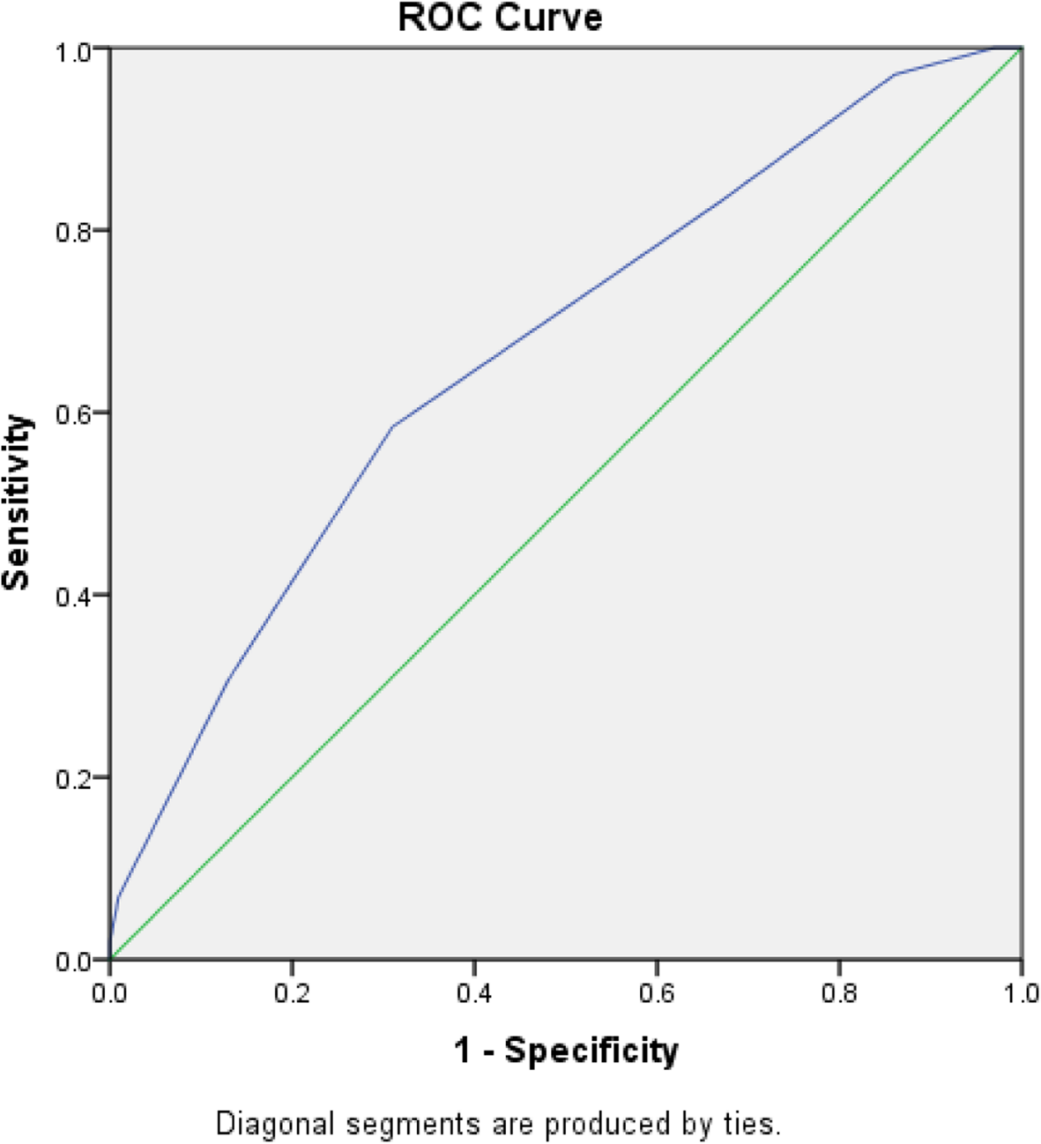
The role of the ISL1, NFATc1, VEGF, HEY2, TBX5 and MTHFR genes in the cardiovascular system** (**SNPs selected for this study in black**). **Reference: Study of variants associated with ventricular septal defects (VSDs) highlights the unique genetic structure of the Pakistani population

To determine whether GRS can discriminate between cases and controls, we conducted Receiver Operator Curve (ROC) analysis. This is a plot of the true positive rate against the false positive rate. The ROC was well segregated between cases and controls (Fig. 3). Green line is standard line, separating the area in half. While the blue line representing the area under the curve AUC. The curve was above the diagonal line (blue line) and the area under the curve was 0.668 (0.593–0.742) which was statistically significant (*p* < 0.001).

We also compared the combined GRS of five SNPs between cases and controls. It was significantly higher in the cases as compared to the controls. For cases, the mean GRS score was 3.78 ± 1.285 while in controls it was 2.95 ± 1.290 (*p-*value: 0.479, CI: 0.474–1.190). Comparison of GRS quartiles between cases and control showed that the mean GRS of cases was 1.90 ± 0.480 while in controls that was 1.68 ± 0.490 (*p-*value: 0.001, CI: 0.086–0.354). Four GRS quartiles were made: 1 (0–2 risk alleles), 2 (3–5 risk alleles), 3 (6–8 risk alleles) and 4 (9–10 risk alleles). The result of binary logistic regression in different quartiles is shown in Table 4. Quartiles number 3 with risk alleles 6–8 was significantly prevalent in cases in comparison to controls. (8% vs 1% with *p-*value: 0.069). None of the patients had > 9 risk allele (quartile 4) (Table 4).

**Table 4:** Comparison of GRS quartiles between cases and controls

Genetic risk score quartiles	Allele Ranges	Cases	Controls	*P-*value
1	0–2	16%	33%	0.026
2	3–5	76%	66%	0.358
3	6–8	8%	1.0%	0.069
4	9–10	0.00	0.00	––-

## Discussion

A generally accepted concept is that congenital heart disease is a multifactorial genetic anomaly [17]. There are two hypotheses in support of this concept. Firstly, the existence of many causes of congenital heart disease in the general population and secondly the genetic and environmental factors playing a crucial role in etiology of this disease [18]. Genetic analysis of complex disorders like VSDs in pediatric patients is particularly challenging. Genes selected for the present study including, *ISL1* (a LIM homeodomain transcription factor) [19], *NFATc1* (nuclear factor of activated T cells, cytoplasmic 1 a transcription factor) [20], *VEGF* (Vascular endothelial growth factor) [6], *TBX5* (T-Box transcription factors) [21] and *MTHFR* (a methylenetetrahydrofolate reductase) [13] have previously been reported for an association with VSDs in different studies. Characteristically, the Pakistani population is a unique ethnic group in the region of Asia. They have specific religious, cultural, social habits and restrictions. In the Pakistani population, consanguineous marriages are a prominent factor seen as a significant cause of genetic disorders [22, 23].

The allelic and genotypic frequencies for the selected SNPs (*ISL1*: rs1017, *NFATc1*: rs7240256, *TBX5*: rs11067075, *MTHFR*: rs1801133) revealed that cases had higher MAF compared to controls. On the other hand, *VEGF*: rs36208048 showed no significant MAF differences in cases and controls, a result that contradicts a previous study [24]. For variation in the gene *ISL1*, findings were in contrast to published data [25, 26]. The *VEGF* variant was also studied in the 1000 genome project in 53 different populations. In the 1000 genome project, MAF for this variant was reported at 0.29. For the SNPs in the *NFATc1, TBX5* and *MTHFR* genes, results were consistent with previous studies [27–29]. All the selected SNPs were also reported in the 1000 genome project in more than 50 different populations (Table 5).

**Table 5:** Selected SNPs in 1000 genome browser (studied in different populations)

Gene: SNP	MAF (from Ensemble 1000 genome browser)	Number of populations studied for each SNP
*ISL1*: rs1017	0.41	29
*NFATc1*: rs7240256	0.47	56
*TBX5*: rs11067075	0.09	54
*MTHFR*: rs1801133	0.48	65
*VEGF*: rs36208048	0.29	53

Nonetheless, genetic risk scores have added a greater improvement in the prognosis of various complex diseases particularly cardiovascular disorders [4]. Although, for Pakistani population data is available reporting the use of genetic risk score analysis, the reports are for other diseases including coronary artery disease and obesity [2, 8, 30]. However, the use of GRS in VSD risk analysis has not been reported in Pakistani subjects yet. The present study is a pilot scale study for determining the cumulative power of GRS. We examined SNPs at five different loci to determine their combined effect. The combined gene score was significantly higher among cases in comparison to controls.

By using this approach, we had also found a significant association of higher GRS quartiles with VSD in the study population. For calculating GRS, all loci are assumed to have an equal contribution to the outcome in an additive manner [2, 8]. However, this may not always be the case as some variant have larger/smaller effect size than others. We did not observe the upper most quartile with 9–10 risk allele in all study participants. Genotypes in the last quartile indicate the existence of all risk alleles at the selected loci. None of the individuals in the study population had been detected with this genetic makeup.

Previously no study had been performed on the GRS association with VSD in the Pakistani population. The present study was also limited by the smaller sample size even though it is a first attempt to determine the relationship of GRS and VSD. Due to limited resources, we were also unable to include additional SNPs. However, the results provide the basis for development of a panel of variants that can be clinically useful to estimate a lifetime risk for an individual to develop VSD. In the future, therefore, new studies with a higher number of variants and larger sample size should be performed in this unique population to validate the results of current study.

## Conclusion

The findings of current study suggest that the GRS of the selected loci is associated with VSD in the recruited cohort. The genetic risk score can therefore be more informative about the association between genetic markers and disease compared to single SNPs. However, SNPS should be selected carefully for such studies. This is a pilot scale study with a relatively small sample size and the findings should be validate by replication analyses in a larger sample size and with more number of representative SNPs in genes encoding signaling molecules, transcription factors and structural heart proteins.

## Supplementary Information

**Additional file 1: Supplementary Table 1. **Primer sequences, PCR product size, restriction enzymes and band sizes of SNPs selected for this study (1).

## Data Availability

All data is available with the corresponding author and can be accessed on request. Data is also shared on figShare with citation, Sarwar, Sumbal (2022): Genotype Data.xlsx. figshare. Dataset. 10.6084/m9.figshare.20300235.v1.

## Abbreviations

SNP: Single nucleotide polymorphism
GRS: Genetic risk score
ROC: Receiver operating curve
VSD: Ventricular septal defects
*NFATc*: (Nuclear factor of activated T cells
*VEGF*: Vascular endothelial growth factor
TBOX: T-Box transcription
*ISL1*: ISLET1 (a LIM homeodomain transcription factor)
*MTHFR*: Methylene tetrahydrofolate reductase
GWAS: Genome Wide Association Studies
