# Supplementary material for: Genetic studies in the Pakistani population reveal novel associations with ventricular septal defects (VSDs)
Source: BMC Pediatr. 2023 Feb 9;23:67. doi: 10.1186/s12887-023-03851-3 (PMC9909889; doi:10.1186/s12887-023-03851-3)
Supplement: Supplementary file 1 — Additional file 1: Supplementary Table 1. Primer sequences, PCR product size, restriction enzymes and band sizes of SNPs selected for this study (1). [file 12887_2023_3851_MOESM1_ESM.docx]

**Supplementary Table 1: Primer sequences, PCR product size, restriction enzymes and band sizes of SNPs selected for this study (1)**

| **SNPs** | **Gene** | **Primer sequence** | **PCR product size** | **Restriction Enzyme** | **Restriction Fragment Size** |
| --- | --- | --- | --- | --- | --- |
| rs1017  NG_023040.1:g.16138A>T | *ISL1* | F-CTCTTGGCCTGTCCTGTAGC  R-GCAATGCAAGAGCAAACAAA | 318bp | *DraI* | AT: 201, 117,95 and 22bp  AA: 201 and 117bp  TT:201, 95 and 22bp |
| rs36208048 NG_008732.1:g.3877C>A | *VEGF* | F-AACCCCCATTTCTATTCAG  R-CTGTGGAGTCTGGCAAAA | 278bp | AlwN1 | Wild type  cut at C  146bp  132bp |
| rs7240256 NG_029226.1:g.23449T>C | *NFATc1* | FI- GGTCACATGCAGCAGCGCT  RI- TGACATTTTCCACGCCTGACG  FO-GGTTTGCAGTTAACCTTTTCCCA  RO-AGAAAGCTCCTTCTGGCATAGG | ------ | ------ | 196bp (T allele wild type)  239bp (C allele SNP)  384bp outer primers |
| rs11067075 NG_007373.1:g.51682G>T | *TBX5* | FI- GTGATAAGGAATCAGCCGGGT  RI- GAATCCCAACTGGAAGGAGAC  FO- TCCTGCCTAGGAGACAACAAATA  RO-AGACAATGAGGGGAAGTCAGATA | ------ | ------ | 124bp T allele  99bp (wild type, G allele)  171bp outer primers |
| rs1801133  NG_013351.1:g.14783C>T | *MTHFR* | OF-GCTGTTGGAAGGTGCAAGATCA  OR-GAGTGGGGTGGAGGGAGCTTAT  IF-AGAAGGTGTCTGCGGG  IR-AAAGCTGCGTGATGAAAT | ------ | ------ | 177bp for T allele  230 for C allele  366bp outer primer |

**Optimize salting out method: (2)**

This method uses standard chemicals that can be obtained from any major supplier

• EDTA (0.5 M), pH 8.0: Add 186.1 gr of anhydrous EDTA to 800 ml of distilled water. Adjust pH to 8.0 with NaOH pellets. Make up to 1 liter with distilled water. Autoclave at 15 p.s.i. for 15 min.

• 1 M Tris-HC1, pH 7.6: Dissolve 121.1 gr of Tris base in 800 ml of distilled water. Adjust pH with concentrated HCl. Allow mixture to cool to room temperature before finally correcting pH. Make up to 1 liter with distilled water. Autoclave at 15 p.s.i. for 15 min.

• Preparation of Red blood cell lysis buffer: 0.01 M Tris-HCl pH 7.6, 320 mM sucrose, 5 mM MgC12, 1% Triton X 100. Add 10 ml of 1 M Tris, 109.54 gr of sucrose, 1.01 gr MgC12, adjust pH to 8.0 and finally add 10 ml of Triton X-100 to 800 ml of distilled water, and make up to 1 liter with distilled water. Autoclave at 15 p.s.i. for 10 min. Sugars at high temperature can cause caramelization (browning), which degrades the sugars [5].

• Preparation of Nucleic lysis buffer: 0.01 M Tris-HC1, 11.4 mM sodium citrate, 1 mM EDTA, 1 % sodium dodecyl sulphate (SDS). Take 10 ml of 1 M Tris-HC1 (pH 7.6), 3.75 gr of anhydrous EDTA (pH 8.0), 10 gr SDS, 2.94 gr of sodium citrate, and adjust pH to 8.0. Make up to 1 liter with distilled water. Autoclave 15 min at 15 p.s.i.

• TE Buffer, pH 8.0: Take 5 ml of 1 M Tris-HCl, pH 7.6, 2 mL of 0.5 M EDTA, pH 8, and make up to 1 liter with distilled water. Adjust pH to 8.0 and autoclave 15 min at 15. p.s.i.

• Chloroform prechilled to 4°C.

• Ethanol (100%) prechilled to -20°C.

Procedure of DNA Extraction

Before starting DNA extraction, liquid blood venogects should be shake gently by rotating blood mixer (vortex)

1. Pour 500 µl of blood into a 1.5 ml eppendorf tube and add 1000 µl of red cell lysis buffer.

2. Shake microfuge tube gently (up to homogenizing), then spin for 2 minutes at 7000 rpm.

3. Discard supernatant and repeat steps 1-3 two or three more times to remove haemoglobin. It is important to breakdown the pellet by vortexing and rinses it well in red blood cell lysis buffer in order to clean the white blood cells from residual of haemoglobin.

4. Placing the tube on tissue paper for few seconds downward. Be careful from cross-contamination between different samples.

5. Add 400 µl of nucleic lysis buffer to eppendorf tube. Note: if the pellet formed, you must pipette the pellet up to dissolve it.

6. Add 100 µl of saturated NaCl (5M) and 600 µl of chloroform to eppendorf tube and mix on a rotating blood mixer at room temperature then spin it for 2 minutes at 7000 rpm.

7. Transfer 400 µl of supernatant to a new 1.5 ml tube.

8. Add 800 µl of cold (-20°C) absolute Ethanol and shake it gently then vortex it. DNA should appear as a mucus-like strand in the solution phase.

9. Spin the microfuge tube for one minute at 12000 rpm to precipitate, then discard supernatant carefully and let tube be completely dried in room temperature (Place Eppendorf tube downward on the tissue paper).

10. Add 50µl of TE to it then vortex; keep eppendorf tube of DNA in 4°C or -20°C for later uses. We routinely use about one µl per PCR reaction without adverse effects. DNA can be quantified and diluted to a working concentration at this point or simply use 1 µl per PCR reaction. We expect that the yield of this procedure be 100 to 300 ng/µl, DNA. Using the above method, high quality DNA samples from a sheep population were extracted for gene mapping studies.

1. Sarwar S, Tahir A, Liaqat Z, Naseer S, Seme RS, Mehmood S, et al. Study of variants associated with ventricular septal defects (VSDs) highlights the unique genetic structure of the Pakistani population. Italian Journal of Pediatrics. 2022;48(1):1-8.

2. Gaaib JN, Nassief AF, Al-Assi A. Simple salting-out method for genomic DNA extraction from whole blood. Tikrit J Pure Sci. 2011;16(2):1813-662.
